# The immunosuppressive microenvironment and immunotherapy in human glioblastoma

**DOI:** 10.3389/fimmu.2022.1003651

**Published:** 2022-11-17

**Authors:** Xuehua Zhang, Leilei Zhao, He Zhang, Yurui Zhang, Huanyu Ju, Xiaoyu Wang, Huan Ren, Xiao Zhu, Yucui Dong

**Affiliations:** ^1^ Department of Immunology, Binzhou Medical University, Yantai, China; ^2^ Department of Immunology, Qiqihar Medical University, Qiqihar, China; ^3^ Department of Immunology, Harbin Medical University, Harbin, China; ^4^ Department of Neurology, Hongda Hospital, Jinxiang, China; ^5^ School of Medicine, Southern University of Science and Technology, Shenzhen, China; ^6^ School of Computer and Control Engineering, Yantai University, Yantai, China

**Keywords:** glioblastoma, tumor microenvironment, immunosuppressive cells, gut microbiota, immunotherapy

## Abstract

Glioblastoma multiforme (GBM) is the most malignant intracranial tumor in adults, characterized by extensive infiltrative growth, high vascularization, and resistance to multiple therapeutic approaches. Among the many factors affecting the therapeutic effect, the immunosuppressive GBM microenvironment that is created by cells and associated molecules *via* complex mechanisms plays a particularly important role in facilitating evasion of the tumor from the immune response. Accumulating evidence is also revealing a close association of the gut microbiota with the challenges in the treatment of GBM. The gut microbiota establishes a connection with the central nervous system through bidirectional signals of the gut–brain axis, thus affecting the occurrence and development of GBM. In this review, we discuss the key immunosuppressive components in the tumor microenvironment, along with the regulatory mechanism of the gut microbiota involved in immunity and metabolism in the GBM microenvironment. Lastly, we concentrate on the immunotherapeutic strategies currently under investigation, which hold promise to overcome the hurdles of the immunosuppressive tumor microenvironment and improve the therapeutic outcome for patients with GBM.

## 1 Introduction

The tumor microenvironment (TME) facilitates the fusion of tumor cells with the surrounding environment by promoting tumor invasion, angiogenesis, and the secretion of cytokines, thus playing an extremely important role in tumor progression. Glioblastoma multiforme (GBM) is characterized by rapid growth and molecular heterogeneity, along with resistance to treatment, leading to inevitable recurrence (1). Since primary brain tumors generally cannot transfer to other parts of the body owing to the blood–brain barrier, these tumors have a distinct TME from that of other tumors (2). The GBM microenvironment is a highly heterogeneous dynamic system; in addition to GBM cells, the TME contains a series of nonneoplastic cells and related molecules, including infiltrating and resident immune cells such as glioma-associated macrophages (GAMs), as well as matrix components, soluble factors, and extracellular matrix (ECM) (3, 4). The cellular composition of the TME and the accessibility of immune cells vary according to the GBM subtype and the clinical characteristics of individual patients. These factors in turn contribute to the formation of an immunosuppressive GBM microenvironment, which leads to the failure of immunotherapy (5).

As the severe immunosuppressive effect in the TME is one of the characteristic features of gliomas, we here provide an overview of the multiple nontumor components of the immune system present in the TME, focusing on GAMs and other infiltrating immunosuppressive cells, including regulatory T cells (Tregs) and myeloid-derived suppressor cells (MDSCs), which are considered to have protumor and immunosuppressive effects. These components constitute the large, complex network of the GBM immunosuppressive microenvironment, which is conducive to facilitating the immune escape of GBM cells. Evading immune surveillance is recognized as a landmark event in cancer biology; accordingly, immunotherapy now represents the backbone of cancer treatment in clinical oncology (6). Immunotherapy for GBM has also recently come into the research spotlight, including strategies involving immune stimulation, antibody-mediated immunotherapy, adoptive cell immunotherapy, and vaccines. However, clinical trials have not yet proven the effectiveness of immunotherapy in treating GBM (7).

A variety of microbial communities that are dominant in the gastrointestinal tract have been reported to coexist in humans and mice and are collectively known as the gut “microbiota” (8). The role of the microbiota in the immune system is now well-established (9). The gut microbiota plays a key role in the regulation of systemic diseases and brain function by influencing the development and function of host metabolism and the immune system (10). However, the role of the gut microbiota in the development of GBM requires further exploration.

In this review, we discuss the mechanisms by which immune cells function in the GBM immunosuppressive microenvironment and the interaction between gut microbes and gliomas *via* the gut–brain axis. Since the introduction of immunotherapy in the clinical treatment of tumors has improved the prognosis of some patients with solid tumors, we further review the clinical studies related to GBM immunotherapy, including immune checkpoint inhibitors (ICIs), vaccines, and chimeric antigen receptor (CAR)-T therapies, aiming to bring new hope for GBM patients.

## 2 Immunosuppressive cells in the TME contribute to GBM progression

**Figure 1:**
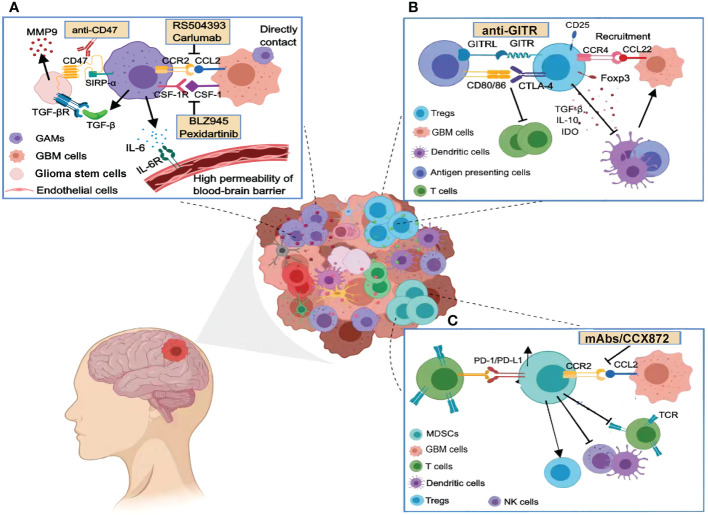
Immunosuppressive cells in the glioma microenvironment. **(A)** GAMs release many cytokines that promote the malignant phenotype of GBM and maintain the high permeability of the blood–brain barrier, including TGF-β, IL-6, and IL-1β. Targeting the phagocytosis checkpoints such as CCL2/CCR2, CD47/SIRP-α, and the CSF-1/CSF-1R axis can enhance the phagocytosis of tumor cells by macrophages. **(B)** Tregs are recruited by chemokines and inhibit the action of cytotoxic T cells through immune checkpoints (e.g., CTLA-4 or GITR). Tregs release immunosuppressive cytokines such as TGF-β, IL-10, and IDO to inhibit dendritic cell function, disturbing a competent anti-tumor immune response. **(C)** MDSCs are also recruited by the CCL2/CCR2 axis in the GBM microenvironment and potently suppress anti-tumor immunity through PD1/PD-L1. MDSCs also deplete human essential amino acids, leading to impaired T-cell activation and function.

GBM creates a local or systemic immunosuppressive microenvironment. Infiltrating immunosuppressive cells account for a large proportion of the GBM microenvironment, and different immunotherapies targeting these immune cells are currently being investigated (**Figure 1**).

### 2.1 GAMs

The macrophages in the GBM microenvironment, or GAMs, are mainly divided into two categories: microglia colonized in the brain (11) and macrophages differentiated from bone marrow-derived monocytes (12). Using genetically engineered mouse models, GAMs were found to be predominantly composed of peripheral macrophages, with a minor population of resident microglia (13). GAMs exhibit marked plasticity and can polarize into M1 and M2 phenotypes with proinflammatory and anti-inflammatory roles under various pathological stimuli (14). M1 GAMs express high levels of the differentiation clusters CD80, CD86, and major histocompatibility complex II (MHC II). M2 GAMs express high levels of CD163, CD206, and CD14; low levels of CD80 and MHC II; and secrete molecules that mediate immunosuppression and promote tumor progression (15). GBM triggers the accumulation of GAMs by regulating chemokines in the TME. M2-directed chemokines are more abundant than M1-directed chemokines in the GBM microenvironment, thereby promoting polarization to the M2 phenotype, and inhibition of the clearance of tumor cells by the M1-type macrophages creates an environment of tumor immunosuppression. The M2 GAMs also promote GBM growth and development, forming a positive feedback regulatory system (16). The engagement of programmed death 1 (PD-1) and its ligand (PD-L1) is an essential mechanism that contributes to the immune-suppressive TME. PD-L1 is highly expressed on tumor-infiltrating myeloid cells (TIMs), including tumor-associated macrophages (TAMs) (17). Zhu et al. (18) reported that TAMs’ infiltration and polarization of M2-type macrophages are both associated with PD-L1–mediated immunosuppression.

GAMs can also directly interact with glioma cells to promote the proliferation of tumor cells. This direct-contact promotion mechanism is related to the increase of Ca^2+^ levels in gliomas, which can transiently stimulate ATP-mediated glioma cells and GAMs. Transforming growth factor (TGF)-β is secreted by GAMs in the TME, which binds to type II TGF-β receptor (TGF-βRII) expressed on the surface of glioma stem-like cells (GSLCs) to promote the secretion of matrix metalloproteinase (MMP)-9 by GSLCs. This mode of action through stimulation of the TGF-β receptor pathway is proposed to be a direct cause of glioma invasion of the surrounding parenchyma (19). Studies have also shown that interleukin (IL)-6 released by GAMs can significantly enhance the permeability of the blood–brain barrier in glioma patients by activating the JAK-STAT3 pathway in endothelial cells and downregulating the level of intercellular connexins, which leads to the formation of vasogenic brain edema (20). IL-1β released by GAMs can also promote the phosphorylation and glycolysis of glycerol-3-phosphate dehydrogenase 2 (GPD2) in glioma cells, thereby accelerating tumor proliferation and growth (21).

### 2.2 Tregs

Tregs are suppressor T cells, which, along with M2-like macrophages/microglia, infiltrate the GBM TME and together constitute the main population of inhibitory immune cells in GBM (22). Therefore, targeting Treg-related mechanisms in GBM patients can improve the success rate of clinical GBM immunotherapy. A correlation between Tregs’ activity and GBM development and immunosuppression has been identified in both mouse models and patients. Tregs represent a subset of CD4^+^ T lymphocytes, which are mainly characterized by high expression of the transcription factors Foxp3, CD25, and cytotoxic T lymphocyte antigen 4 (CTLA-4), with Foxp3 controlling the expression of CTLA-4 in Tregs (23, 24). The number of infiltrating Foxp3^+^ Tregs was found to correlate with the tumor grade. Moreover, Foxp3^+^ Tregs in glioma can bind to CD80/CD86 on antigen-presenting cells (APCs) through CTLA-4, affecting their efficacy and thus inhibiting T lymphocyte activity (25). Tregs infiltrating glioma tissue are significantly more abundant than those in the peripheral blood. Jacobs et al. (26) found that CCL22 secreted by GBM cells could induce the migration of Treg. The CCL22 receptor CCR4 is highly expressed on Tregs in GBM tissues, and other CD4 and CD8 cells in tumor tissues do not express this receptor, suggesting that the recruitment of Tregs in GBM may depend on the action of chemokines.

In GBM-implanted mouse models, the decrease of Tregs led to the proliferation of CD4^+^ T cells and decreased the levels of secreted immunosuppressive cytokines, resulting in tumor rejection and significantly prolonged mouse survival (27). An increase of Tregs was found in GBM compared with the circulation, which may be driven by soluble cytokines produced by GBM. Recent studies have shown that, in addition to priority chemotaxis, soluble cytokines can also induce the proliferation and survival of Tregs. These studies provide new insight into a treatment strategy targeting Tregs (28). Tregs mainly inhibit dendritic cells (DCs), APCs, and other lymphocytes by promoting immunosuppressive factors such as TGF-β, IL-10, and indoleamine-2,3-dioxygenase (IDO), thus creating an immunosuppressive microenvironment (29).

### 2.3 MDSCs

The high accumulation of immunosuppressive cytokines, Tregs, as well as bone marrow-derived inhibitory cells (MDSCs), are important characteristics of the GBM microenvironment (30). MDSCs, identified as CD11b^+^CD33^+^HLA-DR^–/low^ cells, are immature myeloid cells with high heterogeneity that play a key role in tumor cell-induced immunosuppression (31). Patients with GBM were found to have elevated levels of circulating MDSCs, which were 12 times higher than those of healthy individuals (32, 33). MDSCs can be divided into two subsets, including granulocytic MDSCs (G-MDSCs) and monocytic MDSCs (M-MDSCs), which inhibit innate anti-tumor immunity through a variety of mechanisms (34, 35). Data from the study of Bayik et al. (36) demonstrated that the role of MDSCs is sex-dependent in a mouse model of GBM; preclinical models demonstrated that M-MDSCs promoted the progression of GBM in males in the TME, whereas systematic accumulation of G-MDSCs mainly regulated the anti-tumor immune response in females.

There is increasing evidence that the chemokine CCL2 plays a role in the infiltration of MDSCs into the GBM microenvironment. In addition, CCL2 and CCL7 are expressed on GBM and enable CCR2^+^ cells to play a tumor-recruiting role. Loss of CCR2 expression resulted in a reduced outflow of MDSCs in the bone marrow, thereby reducing GBM infiltration of these cells. Other studies demonstrated that CCL2 mediates the migration and accumulation of MDSCs at tumor sites, which not only inhibits the killing function of natural killer (NK) cells and the anti-tumor immune effect of T cells but also promotes the development of Tregs and limits the maturation of DCs, thereby inhibiting innate and adaptive immunity (37–39). MDSCs can use metabolic pathways to mature from bone marrow precursors owing to their high glycolysis flux, and this process indirectly leads to effector T-cell inhibition through the consumption of carbon sources (40). Moreover, MDSCs deplete the availability of human essential amino acids (such as tryptophan, l-arginine, and l-cysteine), leading to downregulation of the TCR-Zeta chain, ultimately resulting in antigen recognition failure and thereby affecting T-cell activation and function (41).

### 2.4 Immunotherapeutic strategies targeting immunosuppressive cells of glioma

#### 2.4.1 Strategies targeting GAMs

GAMS play a critical role in tumor development, and GAM accumulation is correlated with poor survival. Thus, they are an attractive target for GBM immunotherapy. CCL2, which recruits GAMs, may be secreted by GBM cells, and blocking of CCL2’s binding to CCR2 effectively prevents GAM accumulation and increases T-cell and NK cell infiltration (42). Carlumab (CNTO 888), a human IgG1κ anti-CCL2 antibody, was shown that may offer beneficial anti-tumor properties when combined with four chemotherapy regimens in preclinical studies (43). Yang et al. (44) demonstrated, in a mouse xenograft model, that a CCR2 antagonist (RS504393) greatly reduced TAM infiltration and tumor size. Macrophage colony-stimulating factor (M-CSF) and its receptor (CSF1R) are important in both GAM recruitment and differentiation. The usefulness of targeting the binding of CSF-1 and CSF1R was shown in a preclinical study, where a mouse model of ovarian cancer that had been intravenously administered a CSF-1R inhibitor (BLZ945) exhibited a decrease and an increase in TAM cells and CD8^+^ T, respectively (45). Moreover, Omstead et al. (46) showed that pexidartinib, an inhibitor of CSF-1R, inhibited immune escape in solid tumors and enhanced anti-tumor activity; when pexidartinib was combined with a PD-1/PD-L1 inhibitor, CD3^+^CD8^+^ T-cell infiltration increased and M2 macrophage polarization attenuated. CD47 is overexpressed in glioma cells and can block phagocytosis by macrophages. Li et al. (47) found that anti-CD47 antibodies led to increased phagocytosis of glioma cells by macrophages and significantly reduced tumor growth rate in a mouse glioma model. Furthermore, anti-CD47 therapy has been shown to promote the polarization of TAMs from an M2- to an M1-like phenotype (48) and induce anti-tumor effects.

#### 2.4.2 Strategies targeting Tregs

During the early stages of tumor progression, Tregs recruited to the tumor site by the glioma TME can inhibit T-cell functions. In a mouse glioma model, the production of Tregs was found to be time-dependent, and reduction of CD25 expression could inhibit the accumulation of Tregs in the tumor. In addition, the anti-CD25 antibody PC61 caused the specific elimination of CD4^+^CD25^hi^Foxp3^+^ Tregs, resulting in an effective anti-tumor immune response (49). In 2020, Wang et al. (50) found that CD36 expression was upregulated in Tregs and maintained Treg survival through CD36/peroxisome proliferator-activated receptor-β (PPAR-β) signaling. Treatment targeting CD36 resulted in the reduction of intratumoral Tregs and enhancement of the anti-tumor activity of tumor-infiltrating lymphocytes (TILs). Recent studies have demonstrated that, in addition to blocking co-inhibitory pathways, it is also possible to enhance the co-stimulatory pathway to enhance the anti-tumor immune effect. Glucocorticoid-induced tumor necrosis factor (TNF)-related protein (GITR) is a transmembrane protein in the TNF receptor superfamily. Under activation of CD8^+^ and CD4^+^ effector T cells, the expression of GITR was found to rapidly increase and reached the highest level on activated Tregs (51). GITR ligand (GITRL) is mainly expressed by activated APCs. Amoozgar et al. (22) demonstrated that an anti-GITR antibody (αGITR) preferentially targets GBM Tregs by converting immunosuppressed Tregs into anti-tumor CD4^+^ T cells using a preclinical mouse model of GBM. Such immunotherapy strategies targeting GBM-infiltrated Treg-specific phenotypes may be tumor-specific, and the use of Treg-targeted αGITR may reduce immune-related adverse events (52).

#### 2.4.3 Strategies targeting MDSCs

At present, the main therapeutic strategies targeting MDSCs involve the consumption or inhibition of the recruitment of MDSCs or the weakening of the inhibitory activity of MDSCs (53). Kamran et al. (3) found that MDSCs are inhibitors of antigen-specific T-cell proliferation and that interfering with MDSCs enhances the specific CD8^+^ T-cell response induced by TK/Flt3L gene therapy, resulting in an increase in the median survival time and the percentage of mice exhibiting long-term survival. Additionally, the combination of PD-L1 or CTLA-4 inhibitor therapy could greatly improve the therapeutic effect of TK/Flt3L gene therapy. Chemokine receptors are the main driving force for the recruitment of MDSCs. Thus, blocking the binding of chemokine receptors to their ligands can effectively inhibit the aggregation of MDSCs in the TME. For example, monoclonal antibodies targeting CCR2-CCL2 effectively inhibit tumor growth and invasion (43). Flores-Toro et al. (54) reported that the findings of genetic ablation were recapitulated with the use of the CCR2 antagonist CCX872, indicating a reduction of MDSC infiltration in GBM. Moreover, MDSCs can produce polyamines and fatty acids to maintain their immunosuppressive function in GBM. Therefore, inhibition of the production of these substances reduces the survival of MDSCs, thereby activating anti-tumor immunity and impinging the growth of GBM tumors (55).

## 3 Mediating role of the gut microbiota in the immunosuppressive TME

**Figure 2:**
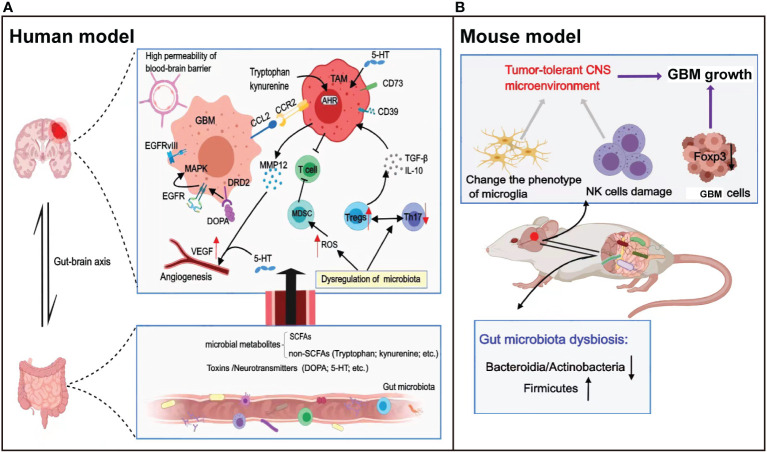
Relationship between the gut microbiota and the development of glioma. **(A)** The gut microbiota drives the production of metabolites and neurotransmitters, which reach the brain through blood circulation and regulate the malignant progression and angiogenesis of GBM *via* direct or indirect effects. In addition, dysregulation of the gut microbiota regulates the expression of ROS or the balance between immune cells to inhibit T-cell killing of tumor cells. **(B)** A glioma-bearing mouse model exhibited gut microbiota dysbiosis with a reduced abundance of Bacteroides and Actinobacteria, and an increased abundance of Firmicutes. In addition, gut microbiota dysbiosis leads to natural killer (NK) cell damage and alters the microglial phenotype, together mediating the tumor tolerance microenvironment in the central nervous system.

Accumulating evidence indicates that the immunosuppressive environment of GBM is not only mediated by the immunosuppressive cells and molecules discussed above but also has many connections with the gut microbiota, thereby promoting the progression of GBM (56). The human gut microbiota contains numerous microorganisms with different properties and functions. Dysbiosis of gut microbiota refers to the inability of bacteria in the human environment to maintain a dynamic balance, leading to inflammation and immunosuppression, and gut microbiota is sensitive to the tumor (57). In recent years, the role of the gut microbiota in tumors has been widely studied, including in gastrointestinal (58), liver, lung, and breast cancers, demonstrating involvement in immune maturation and immune regulation processes (59). However, the mechanism by which the gut microbiota mediates GBM progression remains unclear. In neurodegenerative diseases and tumors of the central nervous system (CNS), the gut microbiota establishes interactions between the gut and the CNS through complex and as-yet-unknown bidirectional signals along the gut–brain axis (60, 61) (**Figure 2**).

Microbiota can regulate local and systemic intestinal immunity, especially in the induction and maturation of immune cells in the nervous system. Studies have reported that gut microbiota dysregulation can downregulate granulocyte-macrophage colony-stimulating factor (GM-CSF) signaling, resulting in activated immature myeloid cells significantly expressing reactive oxygen species (ROS), which increases the inhibitory activity of MDSCs on T cells (62, 63). Moreover, dysregulation of the gut microbiota can affect the immune balance between anti-inflammatory Tregs and proinflammatory Th17 cells (64) and could downregulate the expression of Foxp3 on tumor cells (57), leading to the growth and apoptosis inhibition of glioma cells. Furthermore, a lack of gut microbiota can lead to abnormal immune cell function in the CNS. The morphological characteristics and gene expression profiles of the microglia were altered in germ-free (GF) mice lacking a microbiota, and the increase in the number of immature microglia eventually promoted the progression of glioma (60). D’Alessandro et al. (10) found that gut microbiota dysbiosis led to NK cell damage and altered the microglial phenotype, ultimately impacting the innate and adaptive immune responses of mice. They further established a glioma model by injecting GL261-Luc cells into healthy mice and found that the relative abundance of *Bacteroides* and *Actinobacteria* decreased, while the relative abundance of *Firmicutes* increased, with the progression of glioma.

Specific changes in the gut microbiota and microbial metabolites have been shown to influence disease progression (65). The main metabolites of the gut microbiota are short-chain fatty acids (SCFAs), which activate cellular receptors and affect cellular metabolism (57). SCFAs were shown to ameliorate disease activity by regulating the increase of anti-inflammatory Tregs and the decrease of proinflammatory Th1 and Th17 cells (66). In patients with glioma, metabolites produced by the gut microbiota can affect the immune microenvironment, angiogenesis, and epigenetic landscape through a series of cascade reactions, ultimately influencing the occurrence and development of glioma. More specifically, SCFAs can regulate the levels of TGF-β and IL-10, contribute to the polarization of microglia into M2 phenotype, and inhibit lymphocyte proliferation and T-cell differentiation (63). GBMs are highly vascularized tumors, and glioma growth depends on the formation of new blood vessels. Some studies have reported that bacterial toxins participate in proinflammatory processes and activate angiogenesis (67).

In addition to SCFAs, non-SCFAs produced by gut microbiota metabolism also have a broad regulatory effect on the body. For example, the metabolite tryptophan (Trp) produced by gut microbiota can activate the ligand-activated transcription factor aryl hydrocarbon receptor (AHR), exerting effects on astrocytes, which can regulate nerve excitability and synaptic formation, thereby limiting the occurrence of T-cell–dependent inflammation in the CNS (68, 69). Moreover, glioblastoma cells can produce kynurenine that activates AHR in TAMs; AHR recruits TAMs through CCR2/CCL2, drives the expression of the ectonucleotidase CD39 in TAMs, and plays a synergistic role with CD73 to promote adenosine production, leading to CD8^+^ T-cell dysfunction (70). Gramatzki et al. (71) reported that AHR in glioma cells drives TGF-β expression and that AHR signaling promotes the formation of the immunosuppressive glioma microenvironment.

Neurotransmitters are the products of the activities of the gut microbiota and modulate neuronal activity. D’Alessandro et al. (72) suggested that the ability of the gut microbiota to regulate neurotransmitter levels may be a key factor affecting the progression of brain tumors. In glioma cells, the gut microbiota participated in the regulation of dopamine (DOPA) and serotonin (5-hydroxytryptamine (5-HT)). Studies have reported that dopamine can promote the progression of glioma by binding to dopamine receptor 2 (DRD2), which is highly expressed in GBM cells, activating the expression of epidermal growth factor receptor (EGFR), and promoting the phosphorylation of mitogen-activated protein kinase (MAPK) (73). In addition, the vast majority of 5-HT in the body is produced by gut microbiota metabolism, and the level of secretion determines the degree of anti-tumor and protumor bidirectional effects. Oversecretion of 5-HT can promote the proliferation of gliomas by activating protein phosphorylation signaling pathways (73). It has been previously reported that 5-HT can directly act on adjacent endothelial cells and activate angiogenic pathways (74). Importantly, at the early stages of tumor development, angiogenesis is regulated by 5-HT *via* induction of MMP12 expression in TAMs, thereby decreasing the production of circulating angiostatin (75). The angiogenic effect of 5-HT suggests that it may stimulate cancer cell proliferation and invasion, which are key processes in cancer progression. These studies also demonstrated that 5-HT–activated angiogenic signaling pathways are similar to those activated by VEGF, including the activation of the same signaling kinases, indicating that the downstream angiogenic signaling pathways of VEGF and 5-HT partially converge (74, 76).

Moreover, gut microbes influence the efficacy of cancer immunotherapies, especially ICIs. Vétizou et al. (77) found that the anti-tumor effects of CTLA-4 blocker were related to the presence of different *Bacteroides* species. In tumors treated with antibiotics or in germ-free mice, blocking CTLA-4 had no therapeutic effect, whereas supplementation with *Bacteroides fragilis* significantly enhanced the therapeutic effect. Another study showed that oral administration of *Bifidobacterium* enhanced DC function, leading to CD8^+^ T cells that exerted tumor-killing effects and accumulated in the TME. Combined application of *Bifidobacterium* and PD-L1 checkpoint blockade virtually eliminated tumor growth (78). In addition, with anti-PD-1/PD-L1 therapy, overall survival was higher in patients who did not receive conventional indications of antibiotics compared with that of tumor patients receiving antibiotics, suggesting that disruption of the gut microbiota after antibiotic administration affects the response to immune checkpoint blockade (79). In general, if patients responding to ICIs show a higher abundance of *Faecalibacterium* and *Ruminococcaceae*, the number of CD4^+^ T cells and CD8^+^ T cells will increase, resulting in an overall better anti-tumor effect (80). When stool samples from patients responding to PD-1 blockade were transferred to germ-free mice, the tumor growth rate was significantly reduced, which was attributed to an increase in CD8^+^ T cells and a decrease in Tregs in the TME. In recent years, several studies have emerged to confirm the relationship between the gut microbiota and ICI treatment in patients with several cancers, including nonsmall cell lung cancer, hepatocellular carcinoma, melanoma, and renal cell carcinoma; however, the association in GBM patients still needs to be further explored.

## 4 Immunotherapy for GBM

**Figure 3:**
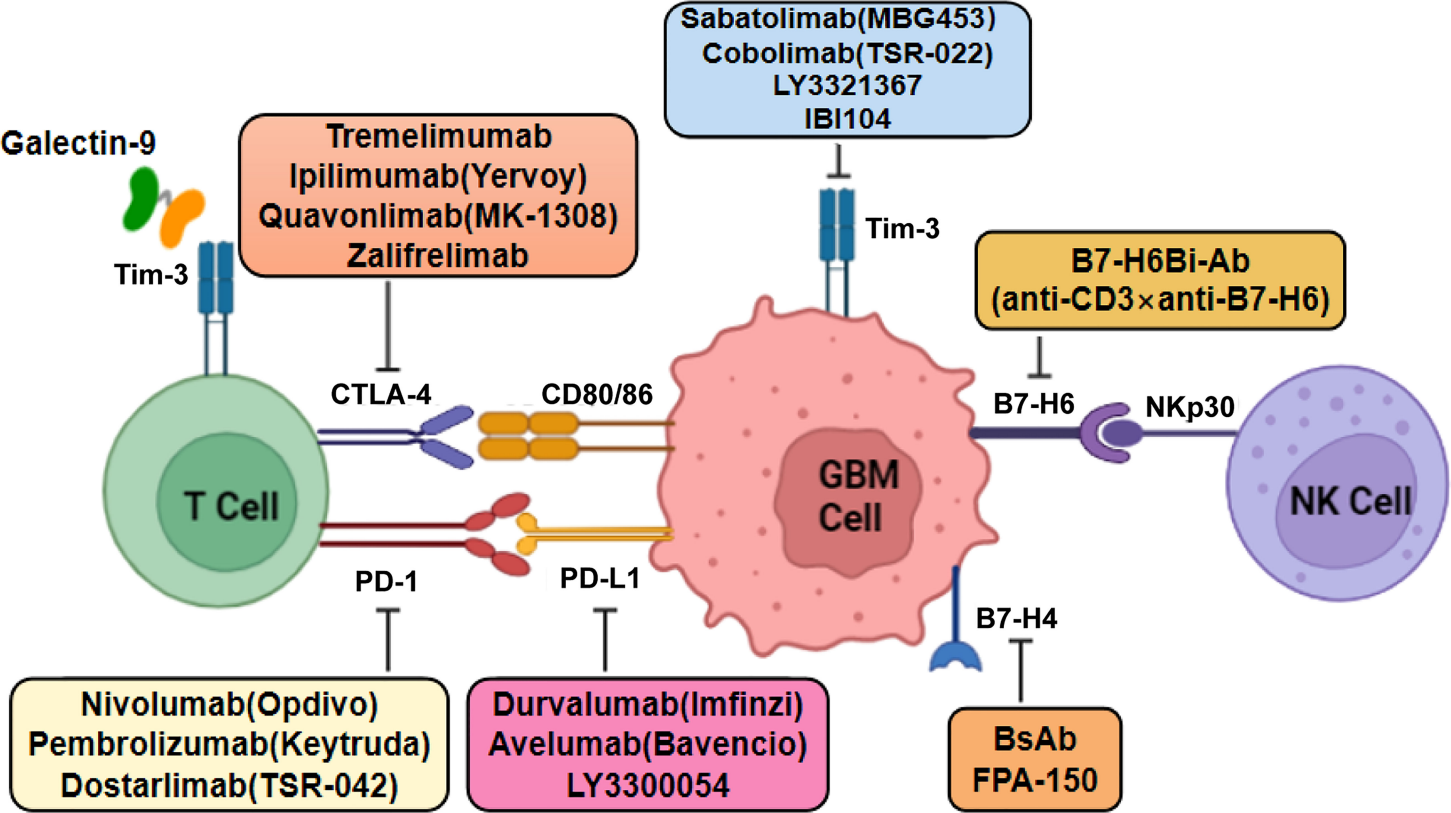
Mechanistic model of the action of monoclonal antibodies against immune molecules in GBM therapy. Classical immune checkpoints such as CTLA-4, PD-1, B7-H6, B7-H4, and TIM-3 bind to their ligands to inhibit T-cell activation and proliferation, thereby creating an immunosuppressive microenvironment. Blocking these immune checkpoint molecules by single or combination therapy with monoclonal antibodies may serve as a potential treatment for glioblastoma.

Tumor immunotherapy is a therapeutic method to control and eliminate tumors by reactivating the tumor-specific immune response and restoring normal anti-tumor immune system activity. With increasing recognition of the immunosuppressive microenvironment created by the persistence of immunosuppressive cells in GBM, clinical treatment is seeing a shift to using ICIs to target immune cell inhibitory receptors (81) (**Figure 3**). Other forms of tumor immunotherapy include passive CAR-T cell immunotherapy and active immunotherapy such as vaccines.

### 4.1 Immune checkpoint inhibitors

#### 4.1.1 CTLA-4 inhibitors

CTLA-4 (CD152) is a negative regulator of T-cell activation, blocking co-stimulatory signals and weakening the function provided by differentiated clusters of CD28 binding B7 (82). Unlike CD28, CTLA-4 is expressed in both activated T cells and Tregs. The affinity between CTLA-4 and CD80/86 was found to be 10- to 20-fold higher than that of CD28, and CD28 was competitively inhibited (83). Therefore, CTLA-4 disrupts the co-stimulatory signaling pathway and inhibits the activation of naive and memory T cells, effectively inhibiting the immune response (84). CTLA-4 inhibitors can block the binding of CTLA-4 with its ligand on the surface of APCs, thereby blocking the inhibitory immune signal and restoring the anti-tumor immune effect of the body. Given the complexity of the GBM immune microenvironment, disrupting CTLA-4/CD80 complex formation in the tumor was found to contribute to the improved survival of GBM-bearing mice (85).

In recent years, CTLA-4 inhibitors have also proven to be successful in tumor immunotherapy in clinical trials. Tremelimumab is a monoclonal antibody targeting CTLA-4 that has shown an effective response in clinical trials when used in combination with a PD-1/PD-L1 inhibitor in various tumors (86, 87). Ipilimumab (Yervoy) is a humanized IgG monoclonal antibody targeting CTLA-4, which showed a clinical benefit in nonsmall cell lung cancer, and its effectiveness in more tumors is currently being evaluated. Its safety and tolerability when combined with other agents, such as temozolomide or radiotherapy, are currently being investigated in phases I and II trials (88). Studies on CTLA-4 immunosuppressants have also shown good effects in patients with glioma. However, further comprehensive analysis of the expression of CTLA-4 in patients is needed to determine the standard drug concentration of CTLA-4 inhibitors in clinical trials (89). Quavonlimab (MK-1308) is a novel humanized immunoglobulin (Ig) monoclonal antibody targeting CTLA-4, thereby preventing the binding of CTLA-4 to CD80/86. The combination of quavonlimab plus pembrolizumab showed a good safety profile in a phase I trial in patients with advanced solid tumors (90). Zalifrelimab (an anti-CTLA-4 antibody) is a novel checkpoint inhibitor, and its combination with balstilimab (an anti-PD-1 agent) in the treatment of recurrent/metastatic cervical cancer has shown promising results. After a two-group phase II study, zalifrelimab was evaluated for safety, tolerability, and efficacy in patients with advanced cervical cancer exhibiting disease progression following chemotherapy (91).

#### 4.1.2 PD-1/PD-L1 inhibitors

PD-1 is expressed in B cells, T cells, MDSCs, TAMs, and NK cells in the GBM microenvironment (92). Among the PD-1 ligands, PD-L1 is highly expressed on the cell surface of a variety of tumor cells, which is related to the immune escape of tumors, whereas PD-L2 is usually only expressed in activated macrophages, DCs, and a few B cells but shows lower expression in tumor tissues. PD-L1 has been more extensively studied than PD-L2 and has become the primary ligand of the immunosuppressive receptor. The binding of PD-1 to PD-L1 forms the immunomodulatory axis, which plays an immunosuppressive role by inducing T-effector cell dysfunction and enhancing Treg function (93). At the same time, the immunomodulatory axis can inhibit the production of numerous cytokines such as ILs and interferon (IFN). PD-L1 has been found to be overexpressed in GAMs and GBM (94, 95). Analysis of a database of clinical samples showed that PD-L1 expression was correlated with the grade of glioma. A higher expression level of PD-L1 in tumor cells was associated with a stronger immunosuppressive effect on T cells, suggesting a new biomarker of glioma (96, 97). Numerous studies have shown that the high level of PD-L1 in the GBM microenvironment is not due to the tumor cells themselves but rather to the abundant myeloid cells such as macrophages infiltrating the tumor (17).

Nivolumab (Opdivo) is a human IgG4 monoclonal antibody that targets PD-1 by binding to the PD-1 receptor, thereby blocking its inhibitory effect. Blocking the binding of PD-1 to its receptor with nivolumab helped boost the T-cell response and restore anti-tumor immunity. Nivolumab has shown efficacy in patients with advanced liver cancer and is currently being tested to expand its use in other populations (98, 99). A National Institutes of Health-led national trial demonstrated that ipilimumab, which targets the CTLA-4 protein, and nivolumab combined with the adjuvant TMZ were safe and tolerable in patients with newly diagnosed GBM. The toxicity of the ipilimumab plus nivolumab combination was not higher than that of the single drug. These data provided necessary safety evidence for subsequent efficacy trials. Phase I of the CheckMate 143 trial (NCT02017717), which was the first to evaluate immune checkpoint inhibition with the first-line treatment of glioblastoma, showed that patients with unmethylated MGMT had similar overall survival with or without TMZ combined with nivolumab plus radiotherapy (100). A randomized phase III study (NCT02667587) demonstrated that nivolumab did not add clinical benefit to standard-of-care radiotherapy plus temozolomide in newly diagnosed GBM with methylated MGMT (101). In another phase III trial (NCT02617589), results showed that the survival of patients with unmethylated MGMT increased more in radiotherapy plus TMZ than in radiotherapy plus nivolumab; therefore, nivolumab was not a suitable replacement for TMZ (102). Pembrolizumab (Keytruda) is another drug targeting PD-1, which is considered to be one of the drugs that helped usher in the era of immuno-oncology (103). In 2019, Cloughesy et al. (104) showed that neoadjuvant pembrolizumab-mediated PD-1 blockade led to an increase of intratumoral T cells, upregulated expression of IFN-γ–related genes, and downregulated expression of cell cycle-related genes, effectively enhancing the local and systemic anti-tumor effect in patients. This mechanism was found to be more effective than adjuvant therapy alone. Durvalumab (Imfinzi) is a high-affinity IgG1 monoclonal antibody that blocks the binding of PD-L1 to PD-1, which has been tested in the treatment of patients with unresectable malignant tumors. Durvalumab showed sustained clinical activity in early clinical trials, both as monotherapy and in combination with tremelimumab (an anti-CTLA-4 agent) (105, 106). Avelumab (Bavencio) is an antibody targeting PD-L1 that has been approved in several countries for the treatment of locally advanced or metastatic urothelial carcinoma (107). Awada et al. (108) reported that avelumab combined with axitinib (a highly selective VEGFR inhibitor) had a synergistic effect in the treatment of recurrent GBM, with the combination having an acceptable toxicity profile.

#### 4.1.3 TIM-3 inhibitors

T−cell immunoglobulin and mucin−domain containing−3 (TIM-3) is considered as a negative regulator of T-cell activation. TIM-3 has been shown to play a role in a variety of diseases, including cancer, by regulating the activity and function of immune cells. TIM-3 is involved in the resistance to PD-1/PD-L1 monoclonal antibodies, and its expression level is increased in mouse models and in patients exposed to anti-PD-1 (109). Galectin-9 is the ligand of TIM-3, and their binding can induce T-cell apoptosis and negatively regulate T-cell immunity. When Th1 cells exert an adaptive immune response, the expression of TIM-3 on differentiated Th1 cells inhibits the Th1 immune response by upregulating galectin-9. In gliomas, TIM-3 can antagonize the inflammatory response and inhibit T-cell–mediated immunity against the tumor (110). Based on RNA-sequencing data from the CGGA Project, Li et al. (111) found that TIM-3 was abundantly expressed in glioblastoma and *IDH*–wild-type glioma with the highest malignant degree. Kim et al. (112) found that TIM-3 was expressed in tumor cells and their surrounding cells (including glial cells and T cells) in an *in situ* mouse glioma model. In TIM-3-mutant mice with intracellular signal transduction deficiency and TIM-3 transgenic mice induced by Cre, TIM-3 affected the expression of immune-related molecules such as iNOS and PD-L1 under exposure to a conditioned medium of primary glial cells from the brain tumor (112). These findings suggested that TIM-3 exerts a positive and unique response to brain tumors and plays an important role in intracellular and intercellular immunoregulation, which differs from its role in the microenvironment of brain tumors.

Several drugs targeting TIM-3 are currently in early-stage clinical trials for different tumor types. Sabatolimab (MBG453) is a humanized IgG4 monoclonal antibody targeting TIM-3, which could block the interaction between TIM-3 and its ligand phosphatidylserine. Sabatolimab also partially blocks the interaction between TIM-3 and galectin-9. In phase I/II trials, sabatolizumab alone or in combination with spartazumab (PDR001, which binds PD-1) was shown to be safe and effective in the treatment of patients with advanced solid tumors (109). A monoclonal antibody targeting TIM-3 (IBI104) blocks the interaction between TIM-3 and phosphatidylserine but not galectin-9. When combined with anti-PD-1, IBI104 shows strong anti-tumor effects *in vivo* (113). Cobolimab (TSR-022), a humanized anti-TIM-3 antibody developed by Tesaro, was found to be safe, which was subsequently tested in combination with dostarlimab (TSR-042; an anti-PD-1 antibody) (114). Another TIM-3 blocking antibody, LY3321367 (Eli Lilly and Company, New York, NY, USA), was found to be successful in early trials. In phase I clinical trials, dose-limiting toxicity was not observed, either alone or in combination with LY3300054, a PD-L1 inhibitor (114).

#### 4.1.4 B7-H4 inhibitors

B7-H4 is a newly identified member of the B7 family (115), which inhibits T-cell proliferation and cytokine secretion. Recent studies found that the B7-H4 protein is highly expressed in tumor tissues but shows low expression in normal tissues, enabling tumor cells to escape attack by the body’s immune system. Yao et al. (116) evaluated the level of B7-H4 in glioma tissue/cerebrospinal fluid among patients with different grades of glioma. They found that the expression level of B7-H4 was related to the prognosis of patients with GBM and was directly related to the degree of malignancy. Glioma initiates the interaction between CD133^+^ cells and Mφs/microglia and activates the expression of B7-H4 in tumor cells and in the TME through IL-6 and IL-10. Chen et al. (117) found that most patients with gliomas expressed PD-L1 or B7-H4; however, few patients showed a high level of co-expression. Patients with high expression of B7-H4 can be regarded as harboring “ultra-cold” gliomas, characterized by a significant lack of TILs, indicating that B7-H4 may inhibit the entry of T cells into the CNS. PD-L1 and B7-H4 thus act as complementary immune molecules in GBM and can be used in immune-targeted or active-specific immunotherapy. The B7-H4 pathway regulating T-cell function and immune escape in patients with GBM is worthy of further exploration for immunotherapy.

Transfection of B7-H4 with small interfering RNA (siRNA) not only reduced the carcinogenicity of the human gastric carcinoma cell line MGC-803 but also induced apoptosis (118). B7-H4 immunoglobulin has been shown to directly regulate the functional level of inflammatory CD4^+^ T cells and is currently under clinical study (119). The B7-H4/CD3 bispecific antibody (BsAb) showed strong anti-tumor activity against B7-H4–positive breast cancer cells and injection of BsAb in humanized mouse models led to the infiltration of CD8^+^ and granzyme B^+^ CTL of tumors. FPA-150 (first-in-class agent developed by Five Prime Therapeutics) is a full-human antibody targeting B7-H4 that blocks the T-cell checkpoint pathway, showing enhanced antibody-dependent cell-mediated cytotoxicity. This is the first therapeutic monoclonal antibody targeting B7-H4 to enter the clinical stage. At present, FPA-150 is in the phase I clinical trial stage, showing a good safety profile when tested as a single drug or in combination with PD-1.

#### 4.1.5 B7-H6 inhibitors

B7-H6 is not expressed in normal human tissues but is highly expressed in human tumor cells. B7-H6 can act as a damage-related molecular pattern to trigger innate immunity (120). B7-H6 was identified as a receptor for NKp-30, an activating receptor for NK cells. The B7-H6–NKp30 complex activates NK cells and kills tumor cells by releasing TNF-α and IFN-γ (121). However, B7-H6 can also be shed from tumors, which may be a mechanism by which tumors evade immune surveillance (122). A study found that B7-H6 and the stem cell marker Sox2 were overexpressed in glioma tissues (123). In addition, B7-H6 was the only gene in the B7 family found to be preferentially expressed in GSLCs. SiRNA-mediated knockdown of B7-H6 inhibited cell proliferation, reduced the expression of the oncogene *Myc*, and inactivated the PI3K/AKT and ERK/MAPK signaling pathways. Lipopolysaccharide-induced expression of B7-H6 and B7-H6 gene knockout inhibited the proliferation, clone formation, migration, and invasion of glioma cells by inducing epithelial–mesenchymal transition-related signal changes (124).

Since B7-H6 is expressed in a variety of malignancies, it is an attractive target for cancer therapy using specific monoclonal B7-H6 antibodies (125). Gacerez et al. (126) constructed CARs based on human single-chain antibodies (scFvs). The results showed that CAR-T cells using human scFvs effectively triggered T-cell effector function when stimulated by tumor cells expressing B7-H6. In addition, human scFv B7-H6–specific CAR-T cells showed different sensitivities to B7-H6 expression on tumor cells and showed effective anti-tumor activity. In the same year, the same group of researchers co-expressed B7-H6–specific CAR and the transcription factor T-bet (T-box expressed in T cells); CD4^+^ T cells were found to enhance the toxicity to B7-H6^+^ tumor cells and improve survival in a RMA/B7-H6 lymphoma mouse model (127). Production of T cells based on the NKp30 chimeric receptor is considered an effective method to detect and treat B7-H6–positive tumor cells (128), which can increase NK cell-mediated tumor destruction and increase the release of bispecific immune oligomeric proinflammatory cytokines (129). Sun et al. (130) constructed bispecific anti-B7-H6 × anti-CD3 (B7-H6Bi antibody-armed T-cells) to target hematological tumors, which showed a significant cytotoxic effect on B7-H6^+^ hematological tumor cells.

### 4.2 CAR-T therapy

Amplification or mutation of *EGFR* occurs in approximately 50% of patients with primary GBM. EGFRvIII, which is the most common consequence of *EGFR*-amplifying gene rearrangement, is expressed only in tumor tissues but not in normal tissues, making it an attractive target for CAR-T therapy. However, in a phase I trial, the third generation of CAR-T EGFRvIII cells derived from human antibodies did not delay the progression or prolong the survival time of patients with recurrent GBM (131). Although EGFRvIII is an attractive target, it has increased the production of antigen-negative escape variants due to its instability. Therefore, overexpression of wild-type *EGFR*, which is found in more than 60% of GBM cases, may be a more attractive target for CAR-T therapy. Choi et al. (132) integrated CART-EGFRvIII with a bispecific T-cell engager (BiTE) that works against EGFR. CAR-T.BiTE cells effectively eliminated heterogeneous tumors in a mouse GBM model. These results suggested that bi-targeted anti-EGFR/EGFRvIII CAR-T cells may be a promising therapeutic strategy in EGFR/EGFRvIII-overexpressing glioblastoma. However, in numerous clinical trials, EGFRvIII-CAR-T has shown many problems. Thus, finding methods to improve the local microenvironment by combining CAR-T and other therapeutic methods has become a research hotspot. A phase I trial of EGFRvIII-CAR-T cells in combination with the anti-PD-1 antibody pembrolizumab was completed last year (NCT03726515).

IL-13 receptor subunit alpha-2 (IL-13RA2) is highly expressed in more than 75% of patients with GBM and is a GBM-restricted receptor associated with a poor prognosis (133). The affinity of IL-13 to IL-13RA2 was found to be stronger than that to IL-13RA1, which inhibits the IL-13RA1/IL-4R signaling pathway (134), suggesting IL-13RA2 as a powerful target for anti-glioma therapy (135). Treatment with IL-13RA2-CAR-T demonstrated a radiographic response of both intracranial and metastatic spinal tumors in patients with multifocal GBM for 7.5 months, and the levels of cytokines and immune cells in the cerebrospinal fluid were correspondingly increased (135, 136). YYB103 is a newly developed CAR-T cell targeting IL-13RA2, which was demonstrated to inhibit tumor growth and prolong the overall survival of U87 MG xenogeneic animal models (137). In addition, transgenic expression of IL-15 is a promising strategy to enhance the effector function of CAR-T cells. IL-13RA2-CAR.IL15 T cells recognize glioma cells, are more proliferative, and produce more cytokines, thus exhibiting more potent anti-tumor activity (138). CAR-T cells targeting IL-13RA2 are currently in phase I clinical trials for ependymoma, GBM, and medulloblastoma (NCT04661384). Moreover, intratumoral delivery of CAR-T cells is being tested in recurrent or refractory malignant glioma (NCT02208362) (139).

Human epidermal growth factor receptor 2 (HER2) is a receptor tyrosine kinase that is a potent immunotherapeutic target for GBM, which is overexpressed in nearly 80% of GBM patients (140). Autologous HER2-CAR-T cells have the ability to kill primary GBM and GBM stem cells and can also induce degeneration in patient-derived xenografts (141, 142). A phase I clinical trial (NCT03500991) of the infusion of HER2-CAR-T cells for the treatment of CNS tumors in children found no dose-limiting toxicity, which resulted in elevated CXCL10 and CCL2 levels in the cerebrospinal fluid (143). Combined with other targets, HER2 is often applied in the study of second- or third-generation CAR-T cell therapy. Given the heterogeneous expression of IL-13RA2 and HER2 in GBM, Hegde et al. (144) hypothesized that a bi-specific CAR molecule, called TanCAR, could target both antigens, which was predicted to eliminate more than 90% of tumors in 20 cohorts of patients with primary GBMs. A recent study reported that HER2-specific CAR-NK cells derived from the human NK cell line NK-92 could effectively kill GBM cells and also showed anti-tumor activity *in vivo* in a mouse model. Currently, HER2-specific CAR-NK cells are in phase I clinical trials (NCT03383978) (139).

### 4.3 Vaccines

Although GBM is associated with many mutation types, EGFRvIII is the only mutant that has been studied as a vaccine target for patients with GBM to date (145). Rindopepimut (CDX-110) is a vaccine developed against EGFRvIII, which was designed by combining an EGFRvIII-specific peptide with keyhole anthocyanin. Phase I/II clinical trials found that overall survival and cessation of steroids were greater than 6 months after treatment in newly diagnosed GBM patients (146). In the phase II clinical trial, the titer of the anti-EGFRvIII antibody increased by approximately four times in 85% of the patients and further increased with the prolongation of treatment time (147). The aim of the phase III clinical trial was to evaluate whether the addition of CDX-110 to standardized treatment could improve the survival of patients with EGFRvIII-mutant GBM, which was terminated after mid-term analysis. In the final analysis, overall survival was not significantly different between the two groups (148).

The novel multipeptide vaccine IMA950 contains 11 tumor-associated peptides (TUMAPs), which have the ability to activate CTLs and limit immune evasion. A phase I trial in GBM patients found that IMA950 was well-tolerated as standardized therapy, with 90% of patients having at least one CD8^+^ T-cell immune response TUMAP and 50% responding to two or more TUMAPs (149). The combination of an IMA950/Poly-ICLC polypeptide vaccine with TMZ in 19 patients (16 with GBM and three with grade III astrocytoma) was confirmed to be safe (150). To date, peptide vaccines have mainly been used for grade IV tumors, but they are slowly being expanded for the treatment of grade II/III gliomas. The nine antigens that make up the IMA950 vaccine were expressed in patients with grade II/III astrocytoma and oligodendroglioma, and the presence of antigen expression and spontaneous immune responses suggested that immunotherapy of grades II and III gliomas could be performed based on the peptide set selected from the IMA950 glioma vaccine (151).

Isocitrate dehydrogenase 1 (IDH1) monoallelic point mutations define a molecularly distinct glioma subtype, with 90% of IDH1 mutations having an arginine-histidine substitution at position 132. IDH1 (R132H) is a potential immunotherapeutic target because it contains an immunogenic epitope suitable for the formation of specific vaccines (152). Previous studies have shown that IDH1-specific peptide vaccines (IDH1-Vac) induce specific therapeutic T helper cell responses and are effective against tumors in IDH1^+^ homologous MHC-humanized mice (153, 154). In a phase I trial (NCT02454634) including 32 patients with grade III/IV glioma, approximately 90% of patients demonstrated an immune response after treatment with an IDH1-R132H^+^–specific vaccine (155, 156). To enhance the efficacy of vaccination, AMPLIFY-NEOVAC (2017-000587-15) proposed combining IDH1 mutation-specific peptide vaccination with PD-L1 checkpoint inhibition to effectively improve therapeutic responsiveness (157).

Heat-shock protein peptide complex-96 (HSPPC-96) is a molecular chaperone of the endoplasmic reticulum and can be ingested by APCs. In a multicenter, open-label phase II trial of 41 adults with surgically resectable GBM who received the HSPP-96 vaccine after total resection, more than 90% of the patients survived for 6 months and nearly 30% survived for 12 months, with a median overall survival of 42.6 weeks (158). Another phase I study (NCT02122822), in which patients with newly diagnosed GBM received the HSPPC-96 vaccine plus standard therapy, found a significant 2.3-fold increase in tumor-specific immune response (TSIR) after vaccination (159). At present, many research centers are exploring the potential of the HSPPC-96 vaccine combined with radiotherapy and chemotherapy in the treatment of primary GBM and the combination of the HSPPC-96 vaccine with bevacizumab in the treatment of recurrent GBM.

In addition to peptide vaccines, autologous formalin-fixed tumor vaccines (AFTV) are undergoing clinical trials as therapeutic agents for glioma. The original method for the preparation of AFTV was developed by Dr. Tadao Ohno (Tsukuba, Ibaraki, Japan). AFTV is prepared using surgically resected formalin-fixed and/or paraffin-embedded patient tumor tissues (160). In the initial clinical trial, 12 patients with primary GBM who were inoculated with AFTV exhibited low expression of p53 and high expression of MHC-I molecules, both of which could significantly improve GBM prognosis (161). Sakamoto et al. (162) described that, in one patient with primary GBM and two patients with secondary GBM, AFTV combined with adjuvant TMZ therapy resulted in a large number of CD3^+^CD8^+^ T cells in surgical specimens. In a prospective phase I/II trial (C000000002), AFTV combined with fractionated radiotherapy (FRT) was used in 24 patients with newly diagnosed GBM: the median overall survival was 19.8 months, and the therapy was well tolerated with low toxicity (163). In another phase I/II trial (UMIN000001426), AFTV and FRT were combined with TMZ adjuvant therapy in patients with newly diagnosed GBM: 33% of the 24 patients had progression-free survival of ≥2 years; the median overall survival was 22.2 months, actuarial 2- and 3-year survival rates were 47% and 38%, respectively, and the therapy was well tolerated (164). A recent case report showed that radiotherapy combined with AFTV therapy resulted in a 91% reduction in tumor volume and maintained regression for 5 years in a patient with brainstem glioma (165). Aruga et al. (166) demonstrated that chemotherapy plus AFTV combined with a peptide vaccine resulted in a strong immune response in patients with biliary tract cancer. However, the combination of AFTV with peptide vaccines or other vaccines in GBM requires further investigation.

## 5 Conclusion

The immunosuppressive microenvironment of GBM facilitates the immune escape of tumor cells and is also an important factor hindering the progress of GBM treatment. Immunosuppression is ultimately the cause of treatment failure for many cancers. Considering the abundance of immunosuppressive cells such as GAMs, Tregs, and MDSCs and their paramount roles in the maintenance of the immunosuppressive TME, we expect such cells to serve as the entry point of targeted treatments to greatly reduce the degree of immunosuppression in GBM. Furthermore, the immunosuppressive environment of GBM has many interrelationships with the gut microbiota, which play an important role in the occurrence, development, and treatment of GBM. Investigating the composition of the gut microbiota and deciphering the gut–immune–brain cancer axis will create further opportunities for the development of effective immunotherapies for malignant brain cancer. There is accumulating evidence that immune cells are inhibited in the glioma microenvironment through a variety of mechanisms, including the presence of immune checkpoints such as PD-1/PD-L1 and CTLA-4. The discovery of immune checkpoints offers new hope for cancer treatment. Peptide- and cell-based vaccines and immunotherapy with immune checkpoint inhibitors are designed to enhance the adaptive immune system with the overall aim to promote a more robust anti-tumor response. In this context, combination therapy targeting complementary mechanisms of action may be required to achieve lasting anti-tumor benefits by improving the GBM immunosuppressive microenvironment.

## Author contributions

XZha and LZ wrote the article and designed the figures. HZ summarized the reference and drafted the manuscript. YZ, HJ, and XW summarized the reference. HR and XZhu revised the manuscript. YD led the study, supervised the overall project, and reviewed the manuscript. All authors contributed to the article and approved the submitted version.

## Funding

This work was supported by the Shandong Provincial Natural Science Foundation of China (ZR2021MH036 to YD), the National Natural Science Foundation of China (61902094 to XZhu and 81472367 to HR), and the Science and Technology Program of Binzhou Medical University (50012304325 to YD).

## Conflict of interest

The authors declare that the research was conducted in the absence of any commercial or financial relationships that could be construed as a potential conflict of interest.

## Publisher’s note

All claims expressed in this article are solely those of the authors and do not necessarily represent those of their affiliated organizations, or those of the publisher, the editors and the reviewers. Any product that may be evaluated in this article, or claim that may be made by its manufacturer, is not guaranteed or endorsed by the publisher.
